# Significantly enhanced lung metastasis and reduced organ NK cell functions in diet-induced obese rats

**DOI:** 10.1186/s40608-017-0161-5

**Published:** 2017-07-03

**Authors:** J. Spielmann, J. Hanke, D. Knauf, S. Ben-Eliyahu, R. Jacobs, G. I. Stangl, I. Bähr, H. Kielstein

**Affiliations:** 1 Department of Anatomy and Cell Biology, Martin Luther University Halle-Wittenberg, Faculty of Medicine, Grosse Steinstrasse 52, 06108 Halle (Saale), Germany; 2 Department of Orthopaedics, Trauma and Reconstructive Surgery, Martin Luther University Halle-Wittenberg, Faculty of Medicine, Ernst-Grube Str. 40, 06097 Halle (Saale), Germany; 3 Neuroimmunology Research Unit, The Sagol School of Neuroscience, The School of Psychological Sciences, Tel Aviv University, 69978 Tel Aviv, Israel; 4 Department of Clinical Immunology and Rheumatology, Hannover Medical School, Carl-Neuberg-Str. 1, 30625 Hannover, Germany; 5 Department of Human Nutrition, Martin Luther University Halle-Wittenberg, Von-Danckelmann-Platz 2, 06120 Halle (Saale), Germany

**Keywords:** Natural killer cells, Obesity, High-fat diet, Diet-induced obesity, Cancer, NKG2D, Tumor cells

## Abstract

**Background:**

Obesity was identified as a major risk factor for malignant diseases, but underlying mechanisms remain unclear. Natural killer (NK) cells, a pivotal aspect of innate immunity, are capable of identifying and killing virally infected and tumor cells. Previous studies have shown altered NK cell functions in obesity, and the current study aimed to investigate the relationship between altered NK cell functions and increased cancer risk in obesity.

**Methods:**

To induce obesity male F344-rats received a high-fat diet (34% fat) or a control diet (4% fat). Thereafter, syngeneic mammary adenocarcinoma cells (MADB106) or a vehicle were intravenously (i.v.) injected. 15 min after injection, half of each group of rats were killed, lungs removed and immunohistochemically stained. Numbers of NK cells, MADB106 cells and NK cell-tumor cell interactions were quantified. Twenty-one days after tumor-cell injection the other half group of rats was killed and lung metastases were counted and relative mRNA concentrations of different NK cell receptors were determined.

**Results:**

After short-term MADB106-challenge, DIO fed animals showed significantly decreased NK cell numbers in the blood and NK cell-tumor cell interactions in the lung as compared to their control littermates. Twenty-one days after MADB106 injection, the lungs of the DIO fed rats showed significantly more lung metastases compared to control animals, accompanied by reduced relative mRNA concentrations of the activating NK cell receptor NKG2D.

**Conclusions:**

We conclude that induction of obesity in F344-rats leads to reduced lung NK cell function against tumor cells and results in significantly enhanced lung metastasis as compared to lean animals. It can be hypothesized that obesity-induced altered NK cell functions play an important role in cancer growth and metastasis.

**Electronic supplementary material:**

The online version of this article (doi:10.1186/s40608-017-0161-5) contains supplementary material, which is available to authorized users.

## Background

Obesity is a worldwide problem, climaxing in the death of 2.8 million people every year [1]. Data of the Global Health Observatory show that in 2014 around 39% of adults aged 18 and over were overweight and 13% were obese [2]. Beside the risk of contracting coronary heart diseases, ischemic stroke, and type 2 diabetes mellitus, common cancers like postmenopausal breast, colon, kidney, esophagus, gallbladder, pancreas and skin cancer [3] are also known to be related to obesity. The underlying biological mechanisms still remain unclear, but there are several studies focusing on the link between obesity and cancer. Besides reduced insulin resistance [4], physical activity [5] and sex hormones [6], also the influence of obesity on immune functions is discussed [7]. Previous studies could show a central role of the adipocytokine leptin on the innate immune system [8–11]. Specifically natural killer (NK) cells seem to be functionally altered by obesity-induced high elevated leptin levels [11–13]. Interestingly, Wrann et al. [12] could show that long-term leptin stimulation significantly impairs NK cell functions such as cytotoxic lysis of tumor cells, interferon γ (IFNγ) secretion, and cell proliferation. NK cells represent 10 to 15% of peripheral lymphocytes and are an essential part of the innate immune system. They play an important role in identifying and killing virally infected and tumor cells without prior sensitization and restriction by major histocompatibility (MHC) antigens [14]. NK cells detect their target cells by surface activating and inhibitory receptors and thereby regulate their activity [15]. After detection, NK cells induce apoptosis of target cells by exocytosis of granzymes and perforin and affect the functions of other immune cells by the release of cytokines, such as IFNγ and tumor necrosis factor α (TNFα) to regulate immune responses [16].

A prospective study by Imai et al. showed that impaired functions of NK cells are associated with an increased incidence for cancer [17]. Additionally, obese patients who lost body weight by bariatric surgery or by exercise training and nutrition courses could reverse their impaired NK cell activity and NK cell-mediated cytokine synthesis [7, 18]. This indicates that there might be a close interaction between impaired NK cell functions in obese individuals and the increased risk for cancer in obesity. Interestingly, there are only a few studies investigating the link of obesity, NK cells and malignant tumor development [19, 20].

Thus, the aim of the present study was to investigate the relationship between altered NK cell functions and increased cancer growth in obesity. F344-rats were fed a high-fat diet to induce obesity. Syngeneic mammary adenocarcinoma cells (MADB106) were i.v. injected to diet-induced obese and normal weight F344-rats to induce lung metastasis. NK cell functions were investigated at two different time points after inoculation of tumor cells. To investigate NK cell functions at an early time point, NK cell-tumor cell interactions and the expression of splenic NK cell receptors were determined 15 min after tumor cell challenge. Furthermore, numbers of lung metastases were determined at a late time point.

## Methods

### Animals and experimental set-up

Male Fischer F344-rats (6-week-old; *n* = 64) were purchased from Charles River GmbH (Sulzfeld, Germany) and kept under controlled conditions at 23 ± 2 °C and 55 ± 5% relative humidity. Rats were maintained on a 12:12 h light dark cycle with lights on from 6 am to 6 pm. Rodent chow and water were available ad libitum. Half of the group (*n* = 32) was randomly selected to receive a high-fat diet (diet-induced obesity: DIO; 29% carbohydrate, 21% crude protein, 35% crude fat and 5.2 kcal/g metabolizable energy; C1090–60 Altromin, Lage, Germany) for 6 (short-term tumor challenge) or 10 weeks (long-term tumor challenge) to induce obesity. Control animals (*n* = 32) received the corresponding control diet (58% carbohydrate, 21% crude protein, 4% crude fat and 3.5 kcal/g metabolizable energy; C1090–10, Altromin). Animals were weighed and handled by the scientists weekly. All research and animal care procedures were approved by the local Animal Care Committee of the “Landesverwaltungsamt Halle” (reference number 42502–2-1116MLU).

After feeding the high-fat diet, DIO animals started the experiments with a significant higher body weight as compared to their age-matched lean littermates.

Subsequently animals were i.v. inoculated with either 1 × 10^6^ cells of an adenocarcinoma syngeneic tumor (MADB106) dissolved in 1 ml isotonic saline or only 1 ml of isotonic saline (NaCl) via the tail vein.

Subsequently half of the animals were killed at an early time point, 15 min after tumor cell challenge (short-term experiment). The other half of the group of rats was admitted to develop lung metastases over a period of 21 days and therefore represent the late time point (long-term experiment). Rats of the long-term experiment received the diet according to their group (control or DIO) for the 21 days of lung metastases development until the end of the experiment.

### Culture and CFSE labeling of MADB106 tumor cells

Cell culture and CFSE (fluorescein derivate 5- (and 6-) carboxyfluorescein diacetate succinimidyl ester)-labeling of cells were conducted as described elsewhere [21]. In brief, the MADB106 mammary adenocarcinoma syngenic tumor is a selected variant cell line obtained from a pulmonary metastasis produced by the intravenous injection of the 9–10 dimethyl-1-2-benzanthracene-induced MADB106 parental adenocarcinoma in F344-rats. Injecting 1 × 10^6^ MADB106-cells via the tail vein of animals leads to pulmonary metastasis after 21 days. For in situ quantification 15 min after injection, tumor cells were vitally dye stained using CFSE (Cell Trace CFSE Cell Proliferation Kit, Molecular Probes, Eugene, USA) before injection [22].

### Blood and organ sampling

Rats were killed under general isoflurane anesthesia either after 15 min (short-term tumor challenge; short-term experiment) or 21 days (long-term tumor challenge; long-term experiment) by puncture of the abdominal aorta. Blood was withdrawn and spleen and liver were removed. Organs were immediately frozen in liquid nitrogen and stored at −80 °C for RNA isolation and lipid analysis. Heparinized blood samples were stored on crushed ice. Subsequently, erythrocytes in the blood were destroyed by a lysis buffer (155 mM NH4Cl, 10 mM KHCO3 and 0.01% EDTA) to obtain leukocytes for following cytometric analysis.

### Lung preparation

Lungs were processed as described by von Hörsten et al. [22]. Briefly, a cannula was inserted into the trachea in situ. Lungs and the heart were dissected from the chest and rinsed with 10 ml 0.9% NaCl. Afterwards, lungs were transfused with 8 ml of O.C.T. embedding medium (Sakura, Tokyo, Japan; diluted 1/5 in PBS) for short-term experiment or with Bouin’s fixative (Sigma-Aldrich, Taufkirchen, Germany) for long-term experiment. Complete lungs were dissected at the hilum from the pulmonary trunk of the heart, frozen in liquid nitrogen and stored at −80 °C for consecutive immunohistochemically staining for the short term experiment or immediately immersion fixed in Bouin’s fixative for 24 h for long-term experiment.

### Immunohistochemically analysis of the lungs (short-term experiment)

Frozen tissue samples of the right lobe were immersed in O.C.T. embedding medium and 5 μm thick sections were cut and placed on glass slides. Every 15th section was selected with a random start, yielding 15–21 sections per animal. Selected sections were mounted on coated glass slides (Starfrost, Knittel, Braunschweig, Germany) and air dried.

Immunostaining of NK cells and CFSE-labeled MADB106 tumor cells was performed using monoclonal antibodies (mAbs) directed against the NK-RP1 receptor (CD 161 rat/ clone 10/78, BioRad AbD Serotec, Puchheim, Germany) on the NK cell surface and the intracellular CFSE antigen (anti-Fluorescein from mouse IgG1 clone B13-DE1, Roche Applied Science, Mannheim, Germany), respectively. Thereafter, APAAP (alkaline-phosphatase-anti-alkaline-phosphatase-complex) staining was performed. Sections were fixed in acetone for 10 min and washed with TBS–Tween (0.05% Tween 20, Serva, Heidelberg, Germany) followed by the incubation with the primary anti-CFSE-mAb overnight at room temperature in humid chambers. Sections were washed with TBS–Tween followed by incubation for 30 min with the bridging antibody (Dako Z 0259, 1/50, rabbit anti-mouse, Dako, Hamburg, Germany) diluted in 5% rat serum. After another rinse the APAAP complex (100 ml Dako D 0651, 1/50, mouse; in TBS–Tween) was added and the sections incubated for 30 min at room temperature. The incubations with bridging antibody and the addition of the APAAP complex were repeated once for 15 min followed by addition of the substrate Fast Blue (Sigma-Aldrich). Next the incubation with the primary antibody against CD161 was performed for 1 h followed by an identical secondary staining procedure except that Fast Red (Sigma) was used as substrate. Finally, sections were counterstained with hematoxylin and covered with glycergel mounting medium (Dako). The immunohistological investigations were carried out strictly under blind conditions. For counting of NK cells and NK cell-tumor cell contacts an area of 40 mm^2^ was evaluated by using the Software Image J software (US National Institutes of Health, Bethesda, MD, USA).

### Visualization of lung metastasis (long-term experiment)

Due to the fixation of lungs in Bouin’s solution 21 days after inoculation of the MADB106 cells, subpleural lung surface metastases were identified by a light, white appearance. Surface metastases are 1–8 mm^3^, mushroom-shaped, distinctly separated, and raised above the lung surface. All visible surface metastases were quantified by masked counting.

### Cytometric analysis

**Fig. 1:**
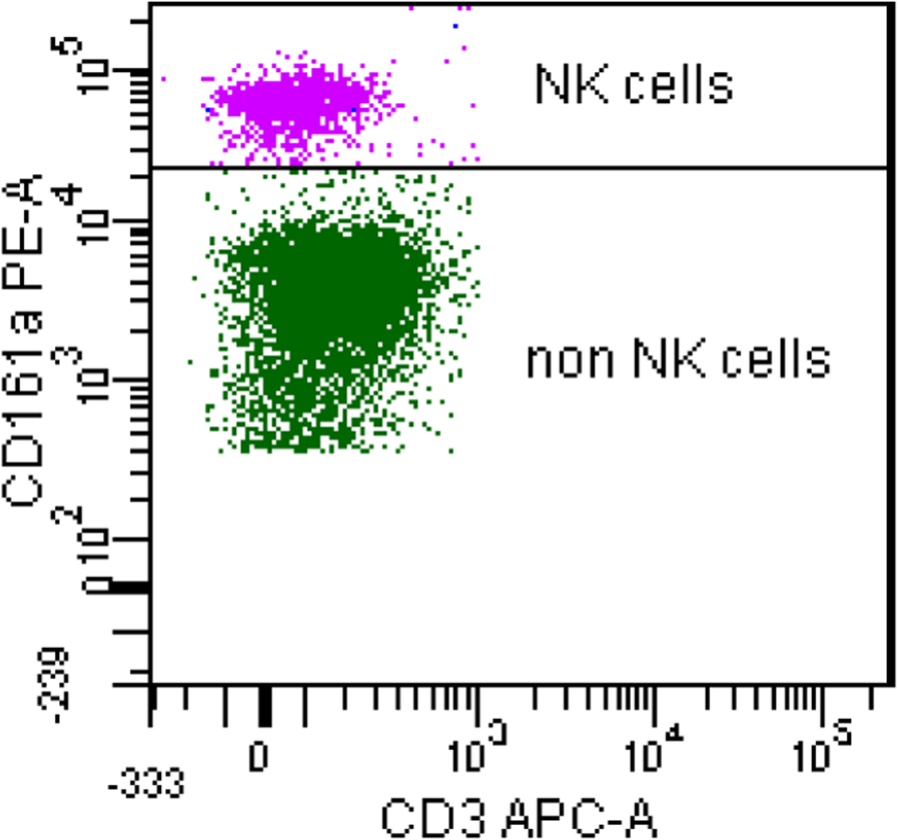
Representative scatter-plot of flow cytometry analysis. NK cells are represented by cells with CD161a^bright^ and CD3^−^ expression and denoted with NK cells

FACS (Fluorescence Activated Cell Sorting) analysis was performed using the following mouse anti-rat mAbs: CD3 conjugated with allophycocyanin (T cell receptor/CD3 APC), and CD161a conjugated with phycoerythrin (NK cells/NKRP1A^+^/CD161a^bright^ PE, BD Biosciences, San Diego, USA). Protected from light, cells were incubated for 30 min at 4 °C. Thereafter, PBMCs were washed twice with washing buffer (PBS supplemented with 1% BSA and 0.1% sodium azide), resuspended in measuring buffer (PBS supplemented with 0.1% BSA and 0.1% sodium azide) and samples were analyzed by flow cytometry using LSR Fortessa with BD FACSDiva Flow Cytometry Software Version 6.2 (BD Biosciences, San Diego, USA; Fig. 1). NK cells are represented by the CD161a^bright^/CD3^−^ population.

### Lipid analysis

Lipids were extracted from liver with a mixture of n-hexane and isopropanol (3:2, *v*/v) as already described [23]. For determination of the concentrations of cholesterol and triacylglycerols (TAG) in the liver, aliquots of the lipid extracts were dried and the lipids dissolved using Triton X-100 (Sigma-Aldrich) [24]. Concentrations of cholesterol and TAG in liver were determined using an enzymatic reagent kit (Ecoline S+, DiaSys GmbH, Holzheim, Germany).

### RT-PCR analysis

Total RNA was isolated by Precellys 24 (Pequlab, Erlangen, Germany) from frozen spleen samples using TRIZOL™ (Sigma Aldrich) according to the manufacture’s protocol. cDNA synthesis was carried out as described by Koenig & Eder [25]. The mRNA concentrations of genes were measured by realtime detection PCR (iQ5, BioRad, München, Germany) using SYBR® Green MIX (BioRad) and the specific primers (KiCqStart™ Primers, Sigma Aldrich, Hamburg, Germany, Additional file 1: Table S1) following the manufacture’s protocol. For determination of mRNA concentration a threshold cycle (Ct) was obtained from each amplification curve using the software Bio-Rad iQ5 (BioRad). Calculation of the relative mRNA concentration was made using the ΔΔCt method [26] with individual amplification efficiency for each primer, determined by a standard curve with different dilutions of primers. The housekeeping gene Cyp18 was used for normalization (Additional file 1: Table S1).

### Statistics

Data analysis was performed using the Graph Pad Prism Software V5 (GraphPad, Inc., La Jolla, CA, USA). For one-way ANOVA Tukey-test and for two-way ANOVA Bonferroni-test with the main factors “diet” and “tumor” were used as post-hoc tests. Means were considered significantly different at *p* ≤ 0.05. Results are presented as means ± standard error of the mean (SEM).

* indicates significant differences of means (*p* ≤ 0.05) between all groups analyzed by one-way ANOVA.

# indicates significant differences of means (*p* ≤ 0.05) between rats receiving NaCl compared to rats receiving MADB106 cells analyzed by two-way ANOVA.

§ indicates significant differences between rats receiving control diet compared to DIO animals (*p* ≤ 0.05) analyzed by two-way ANOVA.

## Results

### Body weight and lipid concentrations in the liver of rats

In both experiments rats fed a DIO diet gained significantly more weight compared to rats fed a control diet (Table 1). In the long-term experiment significant weight gain of DIO-fed animals even continued after MADB106 tumor- cell- injection (Table 1). The concentrations of TAG and cholesterol levels in the liver were significantly higher in animals fed the DIO diet compared to rats fed the control diet (Table 1).

**Table 1:** Body weight, TAG and cholesterol levels in the liver of rats in short-term and long-term experiment

	Short-term experiment	Long-term experiment	
	MADB106 injection	MADB106 injection	21d after MADB106 injection
*Body weight in g*			
Control	290 ± 3	329 ± 4	333 ± 5
DIO	304 ± 4^*^	348 ± 7^*^	363 ± 8^*^
*TAG in the liver in μmol/g*			
Control	38 ± 8		38 ± 4
DIO	90 ± 15^*^		65 ± 9^*^
*Cholesterol in the liver in μmol/g*			
Control	6.7 ± 0.7		7.1 ± 0.3
DIO	11.0 ± 1.6^*^		9.3 ± 0.8^*^

Values are means ± SEM, *n* = 16 rats/group

*TAG* triacylglycerols

^*^Mean values were significantly different from rats fed the control diet: *p* ≤ 0.05

### FACS analysis

**Fig. 2:**
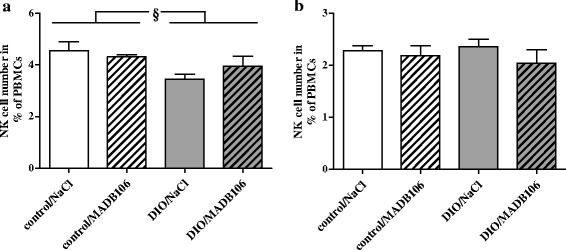
Numbers of NK cells presented as % of peripheral blood mononuclear cells (PBMCs). NK cell numbers are represented by the population with CD161a^bright^ and CD3^−^ expression for (**a**) short-term experiment and (**b**) long term experiment and were determined by cytometry analysis after staining with the appropriate antibodies. Values represent means ± SEM, *n* = 8 rats/group. § indicates significant differences of means between rats receiving control diet compared to DIO fed animals (*p* ≤ 0.05) analyzed by two-way ANOVA

Fifteen minutes after MADB106 cell injection (short-term experiment) DIO fed rats showed significant lower percentage of NK cells of PBMCs compared to rats fed a control diet (Fig. 2). Twenty-one days after the tumor cell challenge (long-term experiment) NK cell numbers of PBMCs was not different between the groups (Fig. 2).

### Relative mRNA concentrations of activating and inhibiting NK cell receptors and cytokines expressed in the spleen

**Fig. 3:**
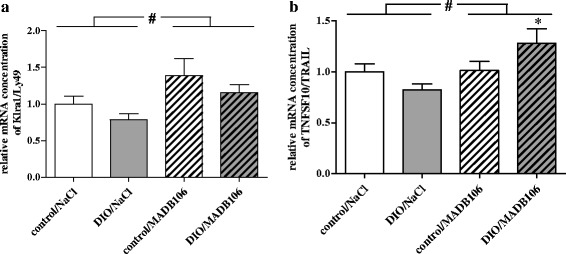
Relative splenic mRNA concentrations of (**a**) Klra1/Ly49 and (**b**) TNFSF10/TRAIL in rats of short-term experiment. Values represent means ± SEM, *n* = 8 rats/group.* indicates significant differences of means (*p* ≤ 0.05) compared to DIO/NaCl analyzed by one-way ANOVA; # indicates significant differences of means (*p* ≤ 0.05) between rats receiving NaCl compared to rats receiving MADB106-cells analyzed by two-way ANOVA

In the short-term experiment (15 min - tumor cell challenge) rats receiving MADB106 tumor cells showed significantly higher relative mRNA concentrations of the inhibiting NK cell receptor Klra1/Ly49 (killer cell lectin-like receptor, subfamily A, member 1) in spleen independently of the dietary regimen (Fig. 3). Relative mRNA concentrations of TNFSF10/TRAIL (tumor necrosis factor (ligand) superfamily, member 10/tumor necrosis factor related apoptosis inducing ligand) of DIO rats receiving MADB106 cells were significantly higher compared to those rats receiving NaCl (Fig. 3). Two-way ANOVA showed a significant influence of MADB106 tumor cell intervention and an interaction with diet on relative mRNA concentrations of TNFSF10/TRAIL in spleens of animals (Fig. 3). Rats did not differ in their relative mRNA concentrations of activating NK cell receptors NCR1/NKp46 (natural cytotoxicity triggering receptor 1), NCR3/NKp30 and Klrk1/NKG2D (killer cell lectin-like receptor k1/natural killer group 2D) and TNFα (Additional file 2: Table S2).

**Fig. 4:**
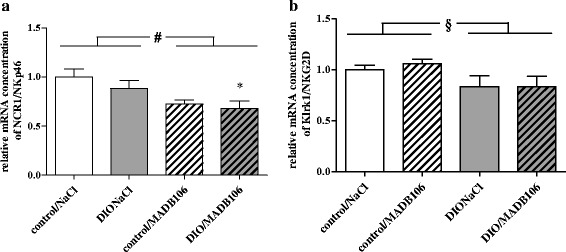
Relative splenic mRNA concentrations of (**a**) NCR1/NKp46 and (**b**) Klrk1/NKG2D in rats of long-term experiment**.** Values represent means ± SEM, *n* = 8 rats/group; * indicates significant differences of means (*p* ≤ 0.05) compared to control/NaCl analyzed by one-way ANOVA; # indicates significant differences of means (*p* ≤ 0.001) between rats receiving NaCl compared to rats receiving MADB106 cells analyzed by two-way ANOVA; § indicates significant differences between rats receiving control diet compared to DIO animals (*p* ≤ 0.05) analyzed by two-way ANOVA

In the long-term experiment (21d - tumor cell challenge) DIO fed rats which developed lung metastases had significantly lower relative splenic mRNA concentrations of the activating NK cell receptor NCR1/NKp46 compared to the corresponding control rats receiving NaCl (Fig. 4). Additionally, two-way ANOVA also showed a DIO independent, significant inhibiting influence of tumor growth on relative mRNA concentrations of the NK cell activating receptor NCR1/NKp46 (Fig. 4). Relative mRNA concentrations of the activating NK cell receptor Klrk1/NKG2D was decreased in rats fed a DIO diet compared to control animals (Fig. 4). Relative mRNA concentrations of NCR3/NKp30, Klra1/Ly49, TNFα and TNFSF10/TRAIL in the spleens of rats did not differ between groups (Additional file 3: Table S3).

### Immunohistochemically quantification of NK cells, MADB106 cells and NK cell-MADB106 cell interactions

**Fig. 5:**
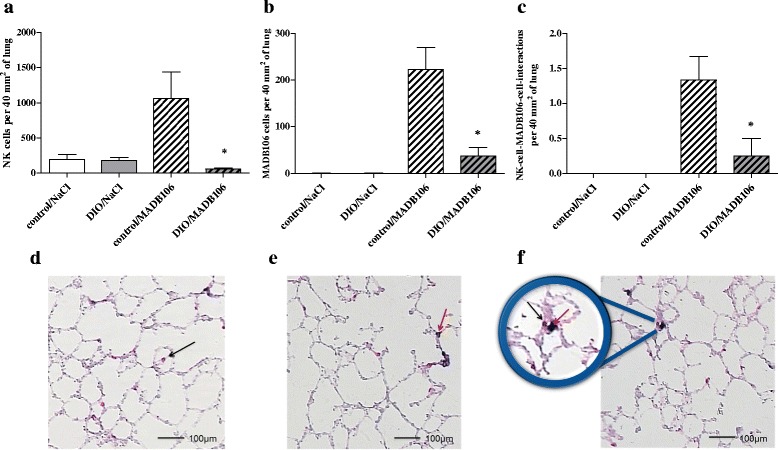
NK cells, MADB106 cells and NK cell-MADB106 cell interactions in lung after short-term tumor challenge. **a**-**c**: Numbers of counted (**a**) NK cells, (**b**) MADB106 cells and (**c**) NK cell-MADB106 cell interactions in the lung after tumor challenge with MADB106 cells or NaCl for 15 min in normal weight (control) or DIO fed rats (DIO); values represent means ± SEM, *n* = 8 rats/group.* indicates significant differences of means (*p* ≤ 0.05) compared to control/MADB106 analyzed by one-way ANOVA. d-f: Double-immunostained and hematoxylin-counterstained cryostat section; staining was performed using anti-CD 161 mAb and anti-Fluorescein mAb, the slices were scanned via digital microscope and analyzed with the ImageJ software: **d** NK cells are stained *red*; **e** MADB106 cells are stained *blue*; **f** NK cell-MADB106 cell interactions; *black arrows mark* NK cells, *red arrows mark* MADB106 cells

In the short-term experiment (15 min - tumor cell challenge) the tissue migration of both tumor cells and NK cells was investigated by immunohistochemistry, with single- and double stained lung sections from animals of all experimental and control groups. DIO-fed rats showed significantly lower numbers of NK cells, MADB106 cells and NK cell-MADB106 cell interactions (Fig. 5).

### Quantification of lung metastases

**Fig. 6:**
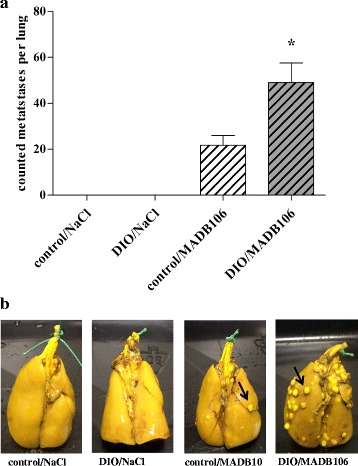
Lung metastasis after long-term tumor challenge. Lungs were fixed in Bouin solution overnight, superficial metastasis appear *white*. **a** Numbers of superficial lung metastases after tumor challenge with MADB106-cells or NaCl for 21 days in normal weight (control) or DIO fed rats (DIO); *n* = 8 rats/group, * indicates significant differences (*p* ≤ 0.05) of means analyzed by one-way ANOVA. **b** Representative photographs of the different lungs after fixation in Bouin solution; *black arrows mark* metastases

In order to quantify and compare the tumor growth between the experimental and control group, lung metastases were quantified 21 days after tumor challenge with MADB106 cells. Rats receiving DIO food developed significantly more lung metastases compared to their lean littermates (Fig. 6).

## Discussion

Obesity is one major risk factor for certain types of cancer [27] and leads to a distinct impairment of various immune cell functions [28, 29]. NK cells are a central component of the innate immune system, secreting different cytokines like IFNγ, interleukin-10 or TNFα to stimulate other immune cells, and are capable to directly destroy tumor cells [16, 30]. NK cells express a wide range of activating and inhibitory surface receptors for recognizing and binding various targets [31]. It has been shown, that the NK cell functionality is significantly impaired in obese individuals [12, 32, 33]. Thus, the present study aimed to investigate effects of obesity on NK cell functions and tumor metastasis after short-term and long-term tumor cell challenge in rats. The first experiment was completed 15 min after tumor challenge and represents the early actions of NK cell defense against tumor cells. The second experiment was terminated 21 days after the tumor challenge and reproduces an advanced state of metastasis under the influence of obesity.

DIO fed rats showed significantly higher values in all three investigated obesity markers (body weight, liver cholesterol and TAG levels) in both experiments.

Results of the short-term tumor challenge showed significantly reduced NK cell numbers in the blood of DIO fed rats compared to the lean littermates. Reduced NK cell numbers could be one reason for the subsequent increased pulmonary metastasis of DIO fed rats, since NK cells play an important role in the early phase of metastasis and fight against tumor cells [34–36], including MADB106 [37]. In this study, 15 min after an intravenous inoculation of MADB106 tumor cells significantly less NK cells, tumor cells and NK cell-tumor cell contacts were found in the lungs of DIO fed rats, suggesting a mechanism that provokes the increased number of lung metastases evident 21 days later. Melemed et al. [38] identified marginating-pulmonary NK cells (MP-NK cells) in the lung capillaries, which showed an increased NK cytotoxicity compared to circulating NK cells and may have a special role in preventing invasion of tumor cells into the lung. In the case of the circulating MADB106 cells in our study, MP-NK cells seem to be influenced by diet-induced obesity, so that in DIO fed animals NK cell numbers in the lung are markedly reduced. In order to evaluate a possible modulation of the NK cell functionality by obesity, we determined the expression of different NK cell receptors. The activity of NK cells is regulated by a balance between activating and inhibiting receptors. Inhibiting NK cell receptors like KIRs in humans and Ly49 in rodents recognize MHC class I molecules and non-MHC ligands and eliminate cells lacking “self” MHC class I molecules according to the “missing self” hypothesis [39–41]. In contrast, activating NK cell receptors, like NKp30, NKp46 and NKG2D, first bind to ligands expressed by tumor cells [42]. In all cases, NK cells exhibit their cytotoxic function after the recognition of target cells by secreting perforin and granzymes from intracellular granules to perforate the cell membrane and induce apoptosis of target cells [43]. Already after a short-term tumor cell challenge (15 min) a significant increase of the expression of the inhibiting Ly49 receptor in the spleen of all tumor cell challenged animals was found, independently of the dietary regimen. It is known that cancer cells can escape immune recognition by upregulating inhibiting NK cell receptors and downregulating activating NK cell receptors [44]. This effect may also be one pathophysiological mechanism for the significantly reduced expression of the activating NK cell receptor NKp46 in all tumor animals 21 days after the tumor cell challenge in the present study. Concerning the late time point, results of the present study showed a significantly reduced expression of the activating NK cell receptor NKG2D in all DIO fed animals. Chung et al. [45] showed a significantly upregulated expression of NKG2D ligands in the adipose tissues of obese mice. In addition O’Rourke et al. [46] found significantly more NKG2D-expressing NK cells in human subcutaneous as well as visceral adipose tissues in obese individuals. Therefore it can be hypothesized that feeding an obesity inducing diet probably induces the upregulation of NKG2D ligands in adipose tissues, thereby recruiting NKG2D receptor expressing NK cells. As a consequence reduced numbers of NKG2D expressing NK cells can be found in the circulation or in other relevant tissues. This may lead to less NK cell-tumor cell contacts in the lung of DIO fed rats, finally resulting in a dramatically enhanced pulmonary metastasis compared to lean animals, as shown in the present study.

Moreover, we recently could show that incubation of the human NK cell line NK-92 with high levels of leptin led to significantly decreased NKG2D expression accompanied with a significant loss of NK cell cytotoxicity against colon cancer cells [20]. As it is known that leptin levels are markedly increased in obese subjects [33, 47, 48] this could be another possible mechanism for the impaired NK cell function and increased cancer risk observed during obesity.

Nevertheless, as the prevalence for obesity [2] and the incidence for obesity related cancers [49] constantly increase, further studies are required to verify these findings and elucidate new mechanisms underlying the influence of obesity on NK cells leading to the observed higher cancer outcome.

## Conclusions

For the first time, a significantly increased lung metastasis in diet-induced obese rodents could be linked to reduced NK cell-tumor cell contacts and a decreased expression of the activating NK cell receptor NKG2D. As NK cells play a major role in tumor cell defense these data provide an important new aspect to elucidate mechanisms underlying obesity-related higher tumor risk. Future studies should further evaluate the molecular mechanisms of a reduced NK cell tissue migration and cytotoxicity in obese compared to normal weight individuals.

## Additional files

Additional file 1: Table S1. Characteristics of the specific primers used for *real-time* RT-PCR analysis. (PDF 19 kb)

Additional file 2: Table S2. Relative mRNA concentrations of NK cell receptors and cytokines in spleen of rats in short-term experiment. (PDF 19 kb)

Additional file 3: Table S3. Relative mRNA concentrations of NK cell receptors and cytokines in spleen of rats in long-term experiment. (PDF 19 kb)

## Abbreviations

APAAP: Alkaline-phosphatase-anti-alkaline-phosphatase-complex
CFSE: Fluorescein derivate 5-(and 6-) carboxyfluorescein diacetate succinimidyl ester
Cyp18: Cyclophilin A
DIO: Diet-induced obesity
IFNγ: Interferon γ
Klra1: Killer cell lectin-like receptor, subfamily A, member 1
Klrk1: Killer cell lectin like receptor k1
MHC: Major histocompatibility complex
NCR1: Natural cytotoxicity triggering receptor 1
NCR3: Natural cytotoxicity triggering receptor 3
NK: Natural killer
NKG2D: Natural killer group 2D
SEM: Standard error of the mean
TAG: Triacylglycerols
TNFSF10: Tumor necrosis factor (ligand) superfamily, member 10
TNFα: Tumor necrosis factor α
TRAIL: Tumor necrosis factor related apoptosis inducing ligand
