# Supplementary material for: Significantly enhanced lung metastasis and reduced organ NK cell functions in diet-induced obese rats
Source: BMC Obes. 2017 Jul 3;4:24. doi: 10.1186/s40608-017-0161-5 (PMC5496225; doi:10.1186/s40608-017-0161-5)
Supplement: Supplementary file 1 — Characteristics of the specific primers used for real-time RT-PCR analysis. (PDF 19 kb) [file 40608_2017_161_MOESM1_ESM.pdf]

Additional table 1. Characteristics of the specific primers used for *real-time* RT-PCR analysis

Gene	Forward and reversed primers	bp	Annealing temperature	NCBI gene bank
**Cyp18**	for: 5’ GTGTTCTTCGACATCACG 3’<br>rev: 5’ AAGTTTTCTGCTGTCTTTGG 3’	92	61°C	NM_017101
**NCR1/NKp46**	for: 5’ AGACCCTGTTTCTTCTCTTG 3’<br>rev: 5’ TGAGCTTCTCATGATCCTTC 3’	197	63°C	NM_057199
**NCR3/NKp30**	for: 5’ AAGAGCCTCCTCAACAAG 3’<br>rev: 5’ AGAAAGCTGAGGGCATAG 3’	94	57°C	NM_181822
**Klra1/Ly49**	for: 5’ CCTGTGTCAACCAAAGAAAG 3’<br>rev: 5’ ATAATTCGCGAAACAGAGAG 3’	181	57°C	NM_001009486
**Klrk1/NKG2D**	for: 5’ TTAATGAGAACAAAGCCTGG 3’<br>rev: 5’ GTTAACTCGTTGGGTGATAG 3’	199	61°C	NM_133512
**TNFSF10/TRAIL**	for: 5’ CTCTACAGTTCCAGAAAAGC 3’<br>rev: 5’ CCTTGGAGATTGGAATTAAGG 3’	128	60°C	NM_145681
**TNFα**	for: 5’ TCTTCTGTCTACTGAACTTCG 3’<br>rev: 5’ AAGATGATCTGAGTGTGAGG 3’	111	56°C	NM_012675

Cyp18 cyclophilin A; **NCR1/NKp46** natural cytotoxicity triggering receptor 1; **NCR3/NKp30** natural cytotoxicity triggering receptor 3; **Klra1/Ly49** killer cell lectin-like receptor, subfamily A, member 1; **Klrk1/NKG2D** killer cell lectin like receptor k1/natural killer group 2D; **TNFSF10/TRAIL** tumor necrosis factor (ligand) superfamily, member 10/ tumor necrosis factor related apoptosis inducing ligand**; TNFα** tumor necrosis factor α
