# Supplementary material for: Significantly enhanced lung metastasis and reduced organ NK cell functions in diet-induced obese rats
Source: BMC Obes. 2017 Jul 3;4:24. doi: 10.1186/s40608-017-0161-5 (PMC5496225; doi:10.1186/s40608-017-0161-5)
Supplement: Supplementary file 2 — Relative mRNA concentrations of NK cell receptors and cytokines in spleen of rats in short-term experiment. (PDF 19 kb) [file 40608_2017_161_MOESM2_ESM.pdf]

Additional table 2. Relative mRNA concentrations of NK cell receptors and cytokines in spleen of rats in short-term experiment

	activating NK cell receptors			***cytokines***
**Groups**	**NCR1/NKp46**	**NCR3/NKp30**	**Klrk1/NKG2D**	**TNFα**
**One-way ANOVA**	*relative mRNA concentrations*			
control/NaCl	1.0 ± 0.1	1.0 ± 0.1	1.0 ± 0.05	1.0 ± 0.2
DIO/NaCl	0.9 ± 0.1	1.1 ± 0.1	0.8 ± 0.1	0.7 ± 0.1
control/MADB106	1.1 ± 0.1	1.1 ± 0.05	1.1 ± 0.1	1.0 ± 0.1
DIO/MADB106	1.1 ± 0.1	1.4 ± 0.3	1.0 ± 0.05	1.1 ± 0.1
**Two-way ANOVA**	*p*			
diet	0.53	0.26	0.21	0.64
MADB106	0.15	0.34	0.09	0.17
diet x MADB106	0.51	0.52	0.33	0.10

Values represent means ± SEM, n=8 rats/group for one-way ANOVA. For two-way ANOVA p-values are shown for the main factors diet, MADB106 and the interaction of both main factors.
