# Supplementary material for: Significantly enhanced lung metastasis and reduced organ NK cell functions in diet-induced obese rats
Source: BMC Obes. 2017 Jul 3;4:24. doi: 10.1186/s40608-017-0161-5 (PMC5496225; doi:10.1186/s40608-017-0161-5)
Supplement: Supplementary file 3 — Relative mRNA concentrations of NK cell receptors and cytokines in spleen of rats in long-term experiment. (PDF 19 kb) [file 40608_2017_161_MOESM3_ESM.pdf]

Additional table 3. Relative mRNA concentrations of NK cell receptors and cytokines in spleen of rats in long-term experiment

	NK cell receptors		***cytokines***	
	activating	***inhibiting***		
**Groups**	**NCR3/NKp30**	**Klra1/Ly49**	**TNFα**	**TNFSF10/TRAIL**
**One-way ANOVA**	*relative mRNA concentrations*			
control/NaCl	1.0 ± 0.1	1.0 ± 0.05	1.0 ± 0.1	1.0 ± 0.1
DIO/NaCl	1.3 ± 0.3	0.9 ± 0.1	1.5 ± 0.3	1.5 ± 0.5
control/MADB106	0.8 ± 0.1	1.0 ± 0.1	1.0 ± 0.2	0.9 ± 0.1
DIO/MADB106	1.1 ± 0.1	0.9 ± 0.1	1.0 ± 0.1	1.0 ± 0.2
**Two-way ANOVA**	*p*			
diet	0.11	0.20	0.21	0.22
MADB106	0.21	0.79	0.22	0.26
diet x MADB106	0.74	0.89	0.29	0.38

Values represent means ± SEM, n=8 rats/group for one-way ANOVA. For two-way ANOVA p-values are shown for the main factors diet, MADB106 and the interaction of both main factors.
